# Three-Dimensional Models of the Oligomeric Human Asialoglycoprotein Receptor (ASGP-R)

**DOI:** 10.3390/ijms11103867

**Published:** 2010-10-11

**Authors:** Ilaria Massarelli, Federica Chiellini, Emo Chiellini, Anna Maria Bianucci

**Affiliations:** 1 UdR INSTM, Department of Pharmaceutical Sciences, University of Pisa, Via Bonanno 6, 56126 Pisa, Italy; E-Mail: ilaria@dcci.unipi.it; 2 Laboratory of Bioactive Polymeric Materials for Biomedical and Environmental Applications (BIOlab)-UdR INSTM, Department of Chemistry & Industrial Chemistry, University of Pisa, Via Vecchia Livornese 1291, 56010 S. Piero a Grado, Pisa, Italy; E-Mails: federica@dcci.unipi.it (F.C.); emochie@dcci.unipi.it (E.C.); 3 Department of Pharmaceutical Sciences, University of Pisa, Via Bonanno 6, 56126 Pisa, Italy

**Keywords:** ASGP-R, oligomeric form, antennary carbohydratic ligands

## Abstract

The work presented here is aimed at suggesting plausible hypotheses for functional oligomeric forms of the human asialoglycoprotein receptor (ASGP-R), by applying a combination of different computational techniques. The functional ASGP-R is a hetero-oligomer, that comprises of several subunits of two different kinds (H1 and H2), which are highly homologous. Its stoichiometry is still unknown. An articulated step-wise modeling protocol was used in order to build the receptor model in a minimal oligomeric form, necessary for it to bind multi-antennary carbohydrate ligands. The ultimate target of the study is to contribute to increasing the knowledge of interactions between the human ASGP-R and carbohydrate ligands, at the molecular level, pertinent to applications in the field of hepatic tissue engineering.

## 1. Introduction

The human asialoglycoprotein receptor (ASGP-R), also called hepatic lectin, is a C-type (calcium dependent) lectin of hepatocytes that recognizes desialylated glycoproteins for endocytosis and lysosomal degradation. It has been largely studied in recent years [1–10] due to its possible role in a wide range of practical applications in human health.

The ASGP-R is an integral membrane protein constituted by four functional domains: a cytosolic N-terminus domain of 40 residues, a single transmembrane domain, a stalk segment of 80 residues and a C-terminal carbohydrate recognition domain (CRD) of 150 amino acid residues. It is able to bind terminal non-reducing galactose and *N*-acetyl-galactosamine residues of desialated tri or tetra-antennary N-linked glycans [1]. The functional form of the human receptor is a noncovalent hetero-oligomer composed of two homologous subunits, generically called H1 and H2 [2].

Simultaneous expression of both subunits was found to be necessary to generate high affinity binding sites. Optimal ligands are triantennary N-linked glycans which bind with a KD in the nanomolar range. Specificity and affinity of ligand binding are accomplished by the simultaneous interaction of at least three terminal ligand residues with three carbohydrate recognition domains (CRDs) [1].

Ligand-receptor cross-linking has revealed that two of the galactose moieties belonging to the ligand specifically interact with H1, and the third one with H2. This finding emphasizes the importance of specific geometric requirements for ligand binding in the spatial arrangement of the CRDs within the receptor complex. Yet, the underlying hetero-oligomeric organization of the subunits is still poorly understood [1].

Simple geometric considerations suggested precise locations of the gal-binding sites of the H1 and H2 subunits; Lodish [9] suggested that they could be arranged so as to generate a triangle of sides 1.5 nm, 2.2 nm and 2.5 nm, at whose vertices are three gal-binding sites for the triantennary oligosaccharide with the highest affinity; two of them should be located on H1 subunits and one on H2.

Moreover, concerning the interactions with the H1 subunits, experimental assays suggested that the two groups of the ligand interact with two different galactose binding sites on two different H1 subunits [11].

These findings infer a few relatively different hypotheses about the 3D arrangement of subunits in the ASGP-R oligomers that give rise to the receptor functional form. In this work we propose a step-wise procedure for building a minimum assessed oligomeric structure, *i.e.*, a H1-H1-H2 trimer. The model obtained proved capable of explaining experimental observations reported in literature and could be used for predictive purposes.

Indeed, the ultimate target of the study is to contribute to increasing the knowledge, at a molecular level, of interactions between the human ASGP-R and carbohydrate ligands with regard to applications in the field of hepatic tissue engineering. In this perspective, hepatic cells seeded on a natural biodegradable carbohydrate scaffold will grow more successfully if the characteristics of the carbohydrate scaffold itself are optimal for scaffold-cells interactions.

## 2. Results and Discussion

### 2.1. Starting Structures

#### 2.1.1. H1 Subunit

In the initial structure for the H1 CRD, taken from PDB (ID: 1DV8, 2.30 Å of resolution), the crystallographic water molecules were removed, except those forming coordination bonds with the Ca^2+^ ions in the binding sites. These water molecules are important since they are replaced by the oxygen atoms of particular hydroxyl groups of the sugar molecule upon binding. The 3D model was subjected to a check of the overall structure and hydrogen atoms were added on the web server MolProbity [12].

The program added H atoms to the initial model only containing heavy atoms, and detected some residues to be flipped (HIS202, ASN208, ASN217, GLN269). The structure was then submitted to geometric analysis. Some warnings were evidenced, the most severe (highlighted in bold in Table 1) refers to rotamer outliers, others, less severe (highlighted in italic), refer to Ramachandran favored and to MolProbity scores. In order to heed these warnings and to remove bad contacts due to the added hydrogen atoms, the structure was submitted to energy minimization by applying the cff91 forcefield implemented in the Discover program (within the InsightII package), with 100 iterations of steepest descent and conjugate gradient until a drms value of 0.001 kcal/Å^2^ was reached.

The minimized structure was submitted to a further geometry check by Molprobity revealing an almost complete resolution of the initial troubles (see Table 1), the warning about rotamers being solved and other indices generally improved. The minimized 1dv8 structure (1dv8_min) was used in the subsequent step of molecular modeling.

#### 2.1.2. H2 CRD Modeling

**Figure 1:**
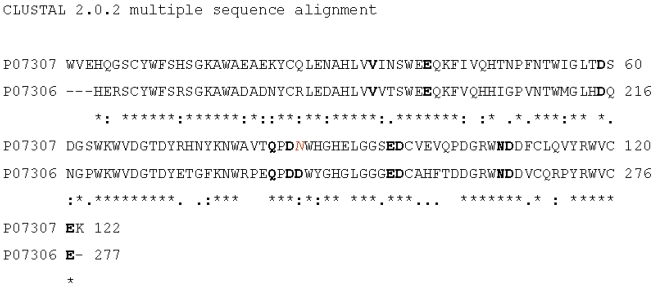
Alignment of the sequences (P07307: human H2 ASGP-R and P07306: human H1 ASGP-R). Only the CRD portions were considered during the alignment. The residue, involved in the coordination bonds of the three binding sites in H1 CRD (evidenced in bold), are perfectly conserved in H2 CRD, except for Asp 242 (belonging to site 1) that in H2 CRD (colored in red) is substituted by a Asn.

3D structure of the CRD belonging to the H2 subunit was obtained (within swisspdbviewer) thanks to its homology with the H1 CRD previously optimized (1dv8_min), and taken as template structure. The homology based modeling protocol relied on a sequence alignment, obtained from the web server Clustalw (as shown in Figure 1), where only the CRD regions were considered, taken respectively from P07307 (human H2 ASGP-R) and P07306 (human H1 ASGP-R) sequences. The alignment score revealed a sequence similarity of about 65%, which ensured that highly reliable models for H2 CRD could be obtained.

The row sequence of CRD of H2 subunit was loaded in swisspdbviewer together the 3D structure of the optimized CRD of H1 (1dv8_min). The alignment, shown in Figure 1, enabled automatic building of a model for H2 CRD, which was then refined on the swissmodel server.

Thanks to the high similarity between the H1 and H2 CRD sequences, the three binding sites (Table 2) identified in the H1 CRD [10] are reasonably conserved in the H2 CRD.

In particular, inspection of the alignment reported in Figure 1, shows that the residues involved in the coordination bonds of the three H1 CRD binding sites are perfectly conserved in H2 CRD with the exception of Asp 242 (belonging to sugar binding site 1) that in H2 CRD is substituted by an Asn residue, which actually possesses very similar chemical properties.

Moreover, the obtained H2 CRD structure was submitted to the web server Q-site Finder [13], a new method for ligand binding site prediction. It works by binding hydrophobic (CH3) probes to the protein, and finding clusters of probes with the most favorable binding energy. Such clusters are ranked by their likelihood of being a binding site, in accordance with the sum total binding energies for each cluster. The results showed that three of the sites predicted by q-site finder (A, B and C in Table 3) include the same residues (highlighted in bold in Table 3), which belong to the three Ca^2+^ binding sites in the H1 CRD.

After ensuring that, in the predicted binding sites of the H2 CRD model, residues corresponding to the ones involved in coordination bonds with Ca^2+^ ions (in the H1 CRD) were included, three Ca^2+^ ions were added in the H2 CRD model, so that the classical coordination geometry of the Ca^2+^ ion with the O atoms of the relevant residues was retained. Moreover, two and three waters molecules were added in sugar binding site 2 and sugar binding sites 1 and 3 respectively, in order to make the coordination geometry of the Ca^2+^ ions to be complete.

The resulting model was then checked on the MolProbity server. Some warnings were found in regard to Ramachandran checks, bad angles and several clashscores. The new model was then submitted to energy minimization, in order to reduce such structural bugs, following a protocol analogous to the ones previously mentioned (100 iteration of steepest descent and then conjugate gradient until a drms value of 0.001 kcal/Å^2^ was reached). In this last case, atoms of the protein backbone and Ca^2+^ ions were allowed to move during energy minimization, while only the distances between each Ca^2+^ ions and their coordinated O atoms were allowed to change in the 3.10–2.10 Å range, in order to retain the coordination bonds. The minimized structure was further submitted to a geometry check on the MolProbity server, revealing a significant improvement compared to the model initially built. The most severe warnings are highlighted in bold (in Table 4) while other less severe ones are highlighted in italic.

The minimized H2 CRD structure (H2_min) obtained by homology from H1 CRD (1dv8_min) was used in the following step of molecular modeling.

#### 2.1.3. Ligands

**Figure 2:**
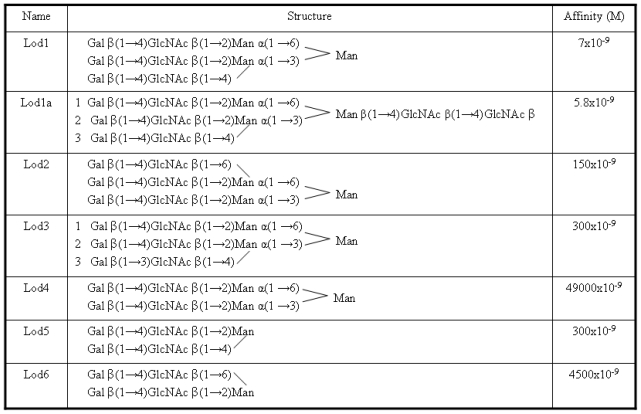
The ligands reported by Lodish [9] and used in this work.

As previously mentioned, the ligands analyzed in this work were taken from an article by Lodish *et al*. [9]. They are reported in Figure 2 with new names, used here for brevity purposes.

A photoaffinity labeling study carried out in rat hepatocytes [11] had shown that a highly ordered binding mode occurs between all three lectin subunits and the three branches of tri-antennary ligands. In particular, for the Lod1a ligand, it was found that the Gal 1 and Gal 2 units bind to the H1 CRD, while the Gal 3 unit binds to the H2 CRD (see Figure 2). Furthermore it has been observed that Gal 1 and Gal 2 bind to different binding site on the two H1 CRDs; Gal 1 should bind to the highest affinity site (site 2) while Gal 2 should bind to another site close to the first one (site 1).

Energy minimization, ligand docking and induced fit studies, which involved the Lod1a ligand, were performed with the aim of reliably assessing the relative locations of the three binding sites (see Table 5).

The three protein subunits were so named on the basis of a trimeric model in complex with the Lod1a ligand. The remaining ligands were similarly allocated in the three binding sites where possible (ligands 1, 3, 4 and 6). Ligands 2 and 5 could give rise to a certain ambiguity. In order to solve this, all plausible starting orientations were sampled and only the best results were considered.

### 2.2. Step-Wise Construction of the Model for a H1-H1-H2 Trimer

The model for the H1s1-H1s2-H2 trimer (where only CRDs were included) was built by following a step-wise procedure. At first, H1s1-H1s2 dimers were built by using three different rigid-body docking programs. Among the H1s1-H1s2 models obtained, the best one was chosen on the basis of different selection criteria, taking into account which one of them fitted to the best experimental binding data involving the ASGP-R and bi-antennary ligands. A further step of rigid-body docking was then performed, so that a H2 CRD unit was added to the selected H1s1-H1s2 dimeric model. The different hypothetical trimeric models obtained were subjected to validation, by applying criteria analogous to the ones mentioned above. The model capable of fitting the best to the affinity trend, shown by bi- and triantennary ligands toward the ASGP-R, was retained as the most plausible model for the H1s1-H1s2-H2 trimer.

#### 2.2.1. H1s1-H1s2 Dimers

The optimized model for H1 CRD (1DV8_min) was subjected to the three rigid-body docking programs Rosetta, HEX, GRAMM in order to obtain different hypotheses for interactions between two H1 CRD monomers. For each program, at least two or three runs were performed changing the initial relative orientation of the two monomers. For each run, the dimeric models were subjected to visual inspection. Only the ones showing orientation between monomers, which appeared to be compatible with available experimental evidence (distances between binding sites, *etc*., as described in more detail later), were selected and retained for subsequent steps of the study.

After the above preliminary selection, other aspects were considered in order to select valid models, for example properties of the monomer contact surfaces were considered. In particular for each proposed dimer, surface number connections, H bonds (true or potential) and salt bridges were counted. Structure 12 and 28 of the HEX run n. 2 had the most interesting results with regard to the contact between single monomers (see Table 6).

##### 2.2.1.1. Energy Minimization

In order to remove bad contacts, the poses found for the H1s1-H1s2 dimer (dimer12 and dimer28) were submitted to a two-step energy minimization protocol carried out by using the cff91 forcefield of Discover, with 100 iteration of steepest descent and conjugate gradient until a drms value of 0.001 kcal/Å^2^ was reached. Distances between each Ca^2+^ ion and its coordinated O atoms in the protein monomers or ligands were allowed to only take values in a range of 3.10–2.10 Å, in order to retain the coordination bonds.

##### 2.2.1.2. Validation of H1s1-H1s2 Dimers

The identification of the most plausible dimeric model from the two previously selected (dimer structures 12 and 28 from the HEX run n. 2), was accomplished by estimating which one of them fitted best the experimental binding data involving ASGP-R and three known bi-antennary ligands (Lod4, Lod5, Lod6).

**Figure 3:**
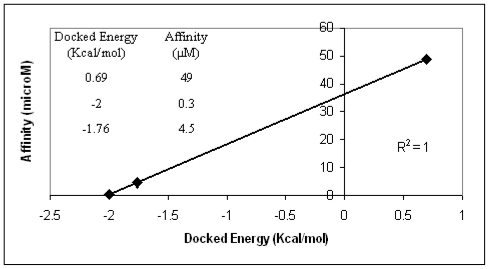
Comparison between AutoDock energies and experimental affinity data of bi-antennary ligands docked in the hypothetic H1s1-H1s1 dimer28.

The AutoDock3 program was used for docking the above ligands into the binding areas of dimer12 and dimer28. The three ligands were not properly allocated by the program when dimer12 was considered. It means that no favorable conformations enabling interactions between each ligand and the two Ca^2+^ ion of interest were found. Optimal ligand conformations interacting with the two Ca^2+^ ions were found, instead, in the case of dimer28. Moreover, docking energy scores obtained for such conformations are in optimal agreement with the experimental affinity data (Figure 3). Based on the above validation check, the model that most realistically fits the experimental affinity trends is dimer28.

#### 2.2.2. H1s1-H1s2-H2 Trimers

After its selection, dimer28 was subjected to the three rigid-body docking programs Rosetta, HEX, GRAMM in order to obtain different hypotheses for the binding surface between the H1s1-H1s2 dimer itself and one subunit of the previously optimized model for H2 CRD. For each program, at least two or three runs were performed changing the initial relative orientation of the partners. For each run, only the trimers showing orientation between monomers compatible with the available experimental evidence (distances between binding sites, *etc*.) were selected by visual inspection and retained. Further checks were performed on the models retained by considering properties of the monomer contact surfaces. In particular for each proposed trimer, surface number connections, H bonds (true or potential) and salt bridges were counted (data not shown).

Among the many different hypotheses generated by the above approach, one (suggested by different HEX and GRAAM runs) was selected as the one closest to what was hypothesized by Lodish [9]. It refers to an oligomeric model where two H1 CRDs (H1s1 and H1s2) and one H2 CRD unit are arranged according to a triangular shape. At its vertice,s three binding sites for galactose moieties of triantennary high affinity oligosaccharides take place. In the selected model the triangular shape hypothesized by Lodish [9] is constituted by the two H1 CRDs (H1s1 and H1s2) and by the sugar site of H2 CRD, usually referred to as site2, which will be labeled thereafter as H2s2.

##### 2.2.2.1. Refinement of the Starting Hypothetical Trimeric Models

For the energy minimization of the starting trimeric model the protocol described in Section 2.2.1.2. was used. Further conditions were applied during the simulation. The distances between Ca^2+^ ions reasonably involved in the binding with carbohydrate ligands for each protein monomer were allowed to change according to values found in the Lodish model [9] as reported below:

- within quite a narrow range around 2.2 nm for H1s1-H1s2;
- within a larger range between 1.5 and 2.5 nm for H1s1-H2s2 and H1s2-H2s2.

**Figure 4:**
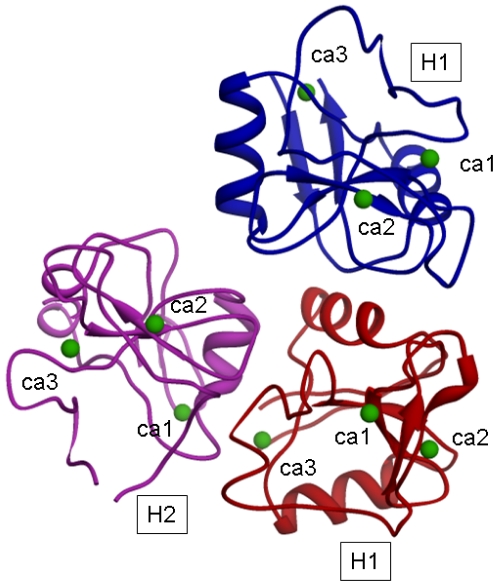
Optimized model of the H1s1-H1s2-H2s2 trimer.

The model for the H1s1-H1s2-H2s2 trimer, after energy minimization, is shown in Figure 4.

The distances (in Å) of Ca^2+^ ions after the minimization are reported in Table 7.

In such a model, a distance of 1.5 nm between H2 and H1 suggested by Lodish could correspond to the distance between the H2 CRD sugar binding site 2 and the H1 CRD sugar binding site 1; moreover the distance of 2.5 nm between H2 and H1 suggested by Lodish could correspond to the distance between the H2 CRD binding site 2 and the H1 CRD binding site 2 (belonging to the H1 subunit of the trimer).

##### 2.2.2.2. Induced Fit

Possible “induced fit” phenomena were analyzed with the aim of estimating the relevance of conformational transitions of the oligomeric receptor upon binding of carbohydratic ligands. The complexes involving the optimized trimeric model and the seven ligands reported by Lodish [9] were further subjected to energy minimization following the protocol described before.

**Figure 5:**
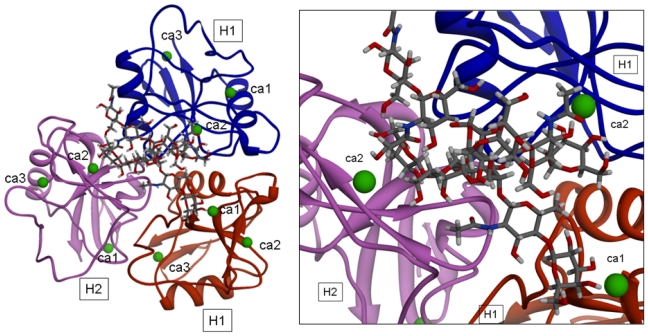
Optimized model of a complex involving the H1s1-H1s2-H2s2 trimer and the Lod1a ligand. Ca^2+^ ions are represented as green spheres

Other than applying the already mentioned restrains on Ca^2+^ atom, the distances between the Ca^2+^ atom of the binding sites (involved in the interaction with the ligands) and the appropriate O atom (belonging to hydroxylic group 3-OH or 4-OH) of the sugars were allowed to change in the same range (3.10–2.10 Å) in order to permit the formation of coordination bonds (displacing the Ca^2+^ coordinate water molecules). The picture of one of the optimized complexes is reported in Figure 5. The structural analysis showed that significant conformational transitions occur in the trimeric model upon ligand binding.

### 2.3. Quantification of Conformational Changes

In order to quantify *induced-fit* effects, some attributes related to interface contacts between the protein monomers upon binding with the ligands, were evaluated through the Protein-Protein Interaction Server [14], accessible on the web. The relevant ligand was removed from each one of the optimized trimer-ligand complexes, so that different superficial attributes were computed for protein interfaces in the trimer. For each one of them, the total contributions due to the three protein interface H1s1-H1s2, H1s1-H2s2, H1s2-H2s2 are reported in Table 8.

When the ligands bind to their biomolecular targets, conformational transitions happen, so that the shape of the binding pocket becomes more complementary to the ligand conformation itself and the structure of the whole complex becomes tighter. The superficial characteristics of the interfaces of the resulting complexes are in some way related to the strength of the binding between the partners involved in the complex itself.

Some of the interaction contributions calculated for the optimized structures (Table 8) for each complex are in good agreement with the experimental affinity data. In Table 8, the agreement of the interface contributions with the ligand-ASGP-R affinity data expressed as pK_D_ is reported in terms of Correlation Coefficient (R^2^). The *Gap volume index* contribution is the one that best correlates with the experimental data (R^2^ = 0.73). The *Gap volume index* supplies a good estimate of interface complementarity [15]. Since Gap volume is dependent on protein size, this feature is computed by normalizing the Gap volume between protein monomers with their interface area:

Gap Volume Index=Gap volume/Interface area

**Figure 6:**
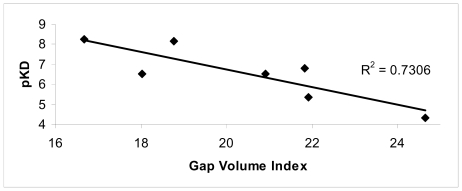
Comparison between Gap volume index values for the optimized complexes and their experimental data affinity (pK_D_).

The smaller the *Gap volume index* is, the more complementary the interface shapes are. Indeed a relationship of inverse proportionality does exist between the *Gap volume index* and the affinity of the ligands for the ASGP-R (Figure 6).

## 3. Methods

### 3.1. Starting Structures

The starting structure for H1 was taken from PDB [16] where the structure of the carbohydrate recognition domain (CRD) of this subunit of the human ASGP-R is available. It was determined via X-ray diffraction at a resolution of 2.30 Å (ID: 1DV8, year: 2000). The corresponding region of the H2 subunit (H2 CRD) was obtained by molecular modeling techniques, based on homology between the two subunits as described in the following sections.

The web server Q-site finder [13] was used to find protein-ligand binding sites. It is an energy-based method and uses the interaction energy between the protein and a simple van der Waals probe to locate energetically favorable binding sites. Energetically favorable probe sites are clustered according to their spatial proximity and clusters are then ranked according to the sum of interaction energies for sites within each cluster.

Ligands and relevant binding data considered in this work are the ones described by Lodish [9] (Figure 2). Their structures were drawn into the Sweet2 web server [17] where a program, enabling the construction of 3D models of saccharides from their sequences expressed through standard nomenclature, is available.

### 3.2. Energy Minimizations

All the energy minimizations were carried out in two steps by means of the Discover program (within the InsightII package [18]), using the cff91 force field with 100 iterations of steepest descent and conjugate gradient until a drms value of 0.001 kcal/Å^2^ was reached. The Ca^2+^ ions were fixed; moreover, distances between each Ca^2+^ ion and its coordinated O atoms in the protein monomers or ligands were allowed to only take values in a range of 3.10–2.10 Å, in order to retain the coordination bonds. Other constrains were imposed for particular cases; they are described in the Results section.

### 3.3. Homology Modeling

The row sequence of the H2 CRD subunit was loaded in the swisspdbviewer [19] together with the 3D structure of the optimized CRD of H1. The alignment was adjusted based on the ones obtained from the ClustalW web server [20]. Then the model of H2 CRD was automatically built and refined by the swiss-model server [19].

### 3.4. Rigid-Body Docking

In order to built plausible H1-H1 dimers, two optimized models of H1 CRD were subjected to the three different “rigid-body” docking programs Rosetta [21], HEX [22] and GRAMM [23], so that different hypotheses for the binding interaction between two H1 CRD monomers could be obtained. For each program, at least two or three runs were performed changing the initial relative orientation of the two monomers. For each run, only the dimers showing orientation between monomers compatible with the available experimental evidence [9] were selected by visual inspection and retained for further steps of the study. After validation (carried out according to what is reported in Section 2.2.1.), a H1-H1 dimer was selected and submitted to other rigid-body docking steps in order to add a CRD model coming from the H2 subunit so that a model for the H1-H1-H2 trimer could be obtained.

#### 3.4.1. Rosetta

The Rosetta package/program was developed at the Baker laboratory of the University of Washington. Its use is free through the web. It works by simultaneous optimization of side-chain conformation and rigid body position of the two docking partners. The former task is performed by a “packing” algorithm, while the latter one is performed by a rigid-body Monte Carlo Minimization (MCM) strategy.

Prior to docking, the sidechains of the native protein are removed and replaced using the Rosetta sidechain packing algorithm to prevent errors in docking due to irregularities (e.g., crystal contacts) in the native protein.

The full atom run can take two forms, depending on one’s confidence in the native structure. Sometimes biochemical and genetic information can be used to localize the binding site to a small region on one or both partners. In this case, one performs a perturbation run, exploring only a small region of space around the suspected binding site. For predictions where there is no biological information about the interface, one usually performs a global search, exploring all the conformational space of both partners [21].

#### 3.4.2. HEX

The HEX program was developed at the Department of Computing Science, University of Aberdeen (UK). Its use is free through the web. In HEX’s docking calculations, each molecule is modeled by using 3D parametric functions which are exploited to encode surface shape, electrostatic charge and potential distributions. The parametric functions are based on expansions of real orthogonal spherical polar basis functions. Essentially, this approach allows each property to be represented by a vector of coefficients. HEX’s surface shape representation uses a novel 3D surface skin model of protein topology, whereas the electrostatic model is derived from classical electrostatic theory. By writing an expression for the overlap of pairs of parametric functions, it is possible to derive a corresponding expression for docking scores as a function of the six degrees of freedom in a rigid body docking search (three translational and three rotational freedom degrees).

With suitable scaling factors, the docking score so obtained can be interpreted as an interaction energy, which may be subjected to minimization. Due to the peculiar orthogonality property of the basis functions, the correlation between a pair of 3D functions (*i.e.*, the overlap expressed as a function of translation/rotation operations) can be computed by means of expressions which only involve the original expansion coefficients. In many respects, this approach is similar to conventional fast Fourier transform (FFT) docking methods based on the use of a Cartesian grid to perform the Fourier transforms. However, the FFT approach only accelerates a docking search in three (translational) degrees of freedom whereas with a spherical polar approach, it is possible to both translate (with some effort) and rotate (relatively easily) the coefficient vectors. Candidate docking orientations are so generated and interaction energies may be estimated in what is effectively a six dimensional Fourier correlation [22].

#### 3.4.3. GRAMM

The GRAMM (Global RAnge Molecular Matching) server was developed at Vakser Lab for protein docking. Its use is free through the web: http://vakser.bioinformatics.ku.edu/resources/gramm/grammx.

This program works thanks to a geometry-based algorithm for predicting the structure of a possible complex between molecules of known structures, by performing an exhaustive six-dimensional search through the relative translations and rotations of the molecules. It can provide quantitative data related to the quality of the contact between the molecules. The intermolecular energy calculation relies on the well established correlation and Fourier transformation techniques exploited in the field of pattern recognition. The docking calculations performed by GRAMM enable predicting the structure of the complex formed between the two constituent molecules by using their atomic coordinates, without any prior information as to their binding sites [23].

### 3.5. AutoDock

The obtained plausible 3D model for the H1-H1 dimer was subjected to a preliminary validation step by estimating its interaction energies with bi-antennary ligands, for which binding data toward the ASGP-R are known. That was achieved by performing flexibile ligand docking studies, by means of the AutoDock program [24].

The starting location of each ligand was manually arranged by approaching the galactose ending moieties of the ligands to the Ca^2+^ ions supposed to interact with the Ca2 site of a H1 subunit and the Ca1 site of the adjacent subunit in the H1-H1 dimer. The two Ca^2+^ ions were named according to Meyer *et al*. [10]. In particular, the 1–6 branches are close to Ca1 and the 1–3 branches are close to Ca2, based on what was suggested by Lodish [9] and Rice *et al*. [11]. The locations of the ligands were subsequently subjected to energy minimization by means of the cff91 forcefield implemented in the Discover program. During the simulations, all atoms of the H1-H1 dimer (whic included Ca^2+^ ions) were fixed, while ligands were allowed to be completely free to move. Spatial restraints were added so that 3-OH and 4-OH atoms of the galactose ending moieties were restrained with respect to the two Ca^2+^ ions within a range of 2–3 Å.

Auto Dock Tools, an accessory program that allows the user to interact with AutoDock from a GUI (Graphic User Interface), was used for preparation of the AutoDock input files. The polar hydrogens and united atom Kollman charges were assigned for the H1-H1 dimer during the preparation of the protein input file, containing fragmental volume and solvation parameters. For the preliminarly optimized ligands, partial atomic charges were determined by a modified Gasteiger method which ensures unit charge on each residue. Moreover, rotatable bonds in the ligands were assigned. Prior to the AutoDock, AutoGrid was carried out for the preparation of the grid map using a grid box with a number of points in xyz (npts) of 40-62-40 Å, which defines the simulation space. The box spacing was 0.375 Å and the grid was set in order to cover the entire space of binding site. A distance-dependent function of the dielectric constant was used for the calculation of the energetic maps.

A scoring grid was calculated from a reference ligand (the one labeled as Lod4 in Figure 1), to minimize the computation time. Finally AutoDock was run using maximum number of energy evaluations retries and generations of 10000 and 27000, respectively. The Lamarckian genetic algorithm (LGA) with the pseudo-Solis and Wets modification (LGA/pSW) method was used with default parameters for calculation of the docking possibilities.

## 4. Conclusions

In the work presented here a homology model of the CRD of the H2 subunit of human ASGP-R was built based its high sequence similarity with the H1 subunit. Then, two H1 and one H2 subunits were added in a step-wise articulated protocol to build the minimal plausible oligomeric form of ASGP-R needed to bind tri-antennary and bi-antennary carbohydratic ligands, that are the most affine (in particular tri-antennary) for such a receptor.

In the first step, dimers H1-H1 were generated by using rigid-body docking programs. Some relevant hypotheses were investigated by comparison with experimental binding data reported in the literature. Furthermore, a docking study was performed using three bi-antennary ligands. It was shown that the docking energies of the ligands in the dimer were in optimal agreement with the experimental affinity data in the case of one of the hypothesized H1-H1 dimers.

Starting from such a validated hypothesis for the H1-H1 dimer, a second step of rigid-body docking was performed in order to add the H2 subunit and build the H1-H1-H2 trimer. Even in this second case, the generated hypotheses were investigated by comparison with experimental binding data reported in the literature. Moreover, potential “induced fit” phenomena were investigated on the best performing H1-H1-H2 trimer. Each of the seven ligands considered in this work were allowed to energy minimize in the starting hypothesis of the trimer.

Significant conformational changes induced on the trimeric 3D theoretical model by the ligands were revealed as we can expect for a proteic receptor responsible for endocitosis.

The conformational changes were evaluated in terms of changes on the monomer interfaces upon ligand binding. Among the superficial contributions evaluated after ligand binding, a good agreement between the Gap volume index and experimental data was observed (R2 = 0.73).

In conclusion, this work gives:

- a 3D theoretical model of the minimal oligomeric structure of ASGP-R required for tri-antennary ligand binding in agreement with the schematic model drawn by Lodish [9].
- information about the conformational and geometric features on carbohydratic ligands required for interaction with ASGP-R.

The knowledge at the molecular level of interactions between the human ASGP-R and carbohydrate ligands is expected to contribute to the progress in the field of hepatic tissue engineering. In this perspective, the selection of optimal scaffolds, made up of natural biodegradable carbohydrates, will enable successful growth of hepatic cells that are expected to positively interact with the scaffold through the ASGP-Rs located of cell surface.

## Figures and Tables

**Table 1:** MolProbity scores and warnings for H1 CRD structures. The most severe and less severe warnings are evidenced in bold and italic, respectively.

		Initial structure (1dv8)	Minimized structure (1dv8_min)	
All-Atom Contacts	Clashscore, all atoms:	16.49 73th percentile*	1.5 99th percentile*	
Protein Geometry	Rotamer outliers	**5.41%**	0.90%	Goal: <1%
	Ramachandran outliers	0.00%	0.00%	Goal: <0.2%
	Ramachandran favored	*95.24%*	*95.24%*	Goal: >98%
	Cβ deviations >0.25 Å	0	*5*	Goal: 0
	MolProbity score	*2.61* 52nd percentile*	1.22 99th percentile*	
	Residues with bad bonds:	0.00%	0.78%	Goal: <1%
	Residues with bad angles:	0.00%	0.00%	Goal: <0.5%

* 100th percentile is the best among structures of comparable resolution; 0th percentile is the worst. Clashscore is the number of serious steric overlaps (>0.4 Å) per 1000 atoms.

**Table 2:** Oxygen atoms of the residues involved in the coordination bonds with Ca^2+^ atoms in H1 CRD binding sites.

Binding site 1	Binding site 2	Binding site 3
Asp 215 O^δ1^	Asp 241 O^δ1^	Glu 196 O^ɛ1^
Asp 215 O^δ2^	Glu 252 O^ɛ2^	Glu 196 O^ɛ2^
Asp 242 O^δ1^	Asp 265 O^δ1^	Glu 277 O^ɛ1^
Glu 252 O	Asp 265 O	Glu 277 O^ɛ2^
Asp 253 O^δ1^	Asn 264 O^δ1^	Val 190 O
Wat3	Wat11	Wat20
Wat10	Wat13	Wat46
Wat14	Asp 215 O^δ1^	Wat84

**Table 3:** Clusters of residues in the CRD H2 structure identified by the Q-site Finder web server as potential binding sites, ranked on the likelihood of being a binding site, according to the sum total binding energies for each cluster. They include the residues (in bold) that form the three Ca^2+^ binding site in H1.

	A	B	C
residues	GLN 88 ↔ **GLN 239**	ASP 64 ↔ **ASP 215**	HIS 9 ↔ HIS 160
	ASP 90 ↔ **ASP 241**	ASN 91 ↔ **GLU 242**	GLN 10 ↔ GLN 161
	TRP 92 ↔ TRP 243	GLY 98 ↔ GLY 249	GLY 11 ↔ GLY 162
	GLU 101 ↔ **GLU 252**	GLY 99 ↔ GLY 250	SER 12 ↔ SER 163
	ASN 113 ↔ **ASN 264**	SER 100 ↔ SER 251	TYR 14 ↔ TYR 165
	ASP 114 ↔ **ASP 265**	GLU 101 ↔ **GLU 252**	VAL 39 ↔ **VAL 190**
	ASP 115 ↔ ASP 266	ASP 102 ↔ **ASP 253**	ASN 41 ↔ ASN 192
		CYS 103 ↔ CYS 254	SER 42 ↔ SER 193
		ASP 115 ↔ ASP 266	GLU 44 ↔ GLU 195
		PHE 116 ↔ PHE 267	GLU 45 ↔ **GLU 196**
		CYS 117 ↔ CYS 268	GLU 126 ↔ **GLU 277**
		LEU 118 ↔ LEU 269	ARG 128 ↔ ARG 179

**Table 4:** MolProbity scores and warnings for H2 CRD structures. The most severe and less severe warnings are evidenced in bold and italic, respectively.

		Initial structure (H2)	Minimized structure (H2_min)	
All-Atom Contacts	Clashscore, all atoms:	**29.11** 16th percentile*	*18.69* 36th percentile*	
Protein Geometry	Rotamer outliers	0.88%	0.88%	Goal: <1%
	Ramachandran outliers	*0.79%*	*0.79%*	Goal: <0.2%
	Ramachandran favored	**92.86%**	*95.24%*	Goal: >98%
	Cβ deviations >0.25Å	0	*1*	Goal: 0
	MolProbity score	*2.40* 53rd percentile*	*2.19* 65th percentile*	
	Residues with bad bonds:	0.78%	0.00%	Goal: <1%
	Residues with bad angles:	**0.78%**	0.00%	Goal: <0.5%

* 100th percentile is the best among structures of comparable resolution; 0th percentile is the worst. Clashscore is the number of serious steric overlaps (>0.4 Å) per 1000 atoms.

**Table 5:** Location of the ending moieties of the Lod1a ligand branches, in each one of the three binding sites. Subunits type and labels of the specific subunit to which the site belongs are indicated.

Lod1a moiety	Subunit type	Binding site	Subunit label
Gal 1	H1	site 2	H1s2
Gal 2	H1	site 1	H1s1
Gal 3	H2	site 2	H2s2

**Table 6:** Properties of monomer contact surfaces (surface connection numbers, true or potential H bonds, and salt bridges) of H1-H1 dimers after rigid-body docking performed with the Rosetta, HEX, and GRAMM programs.

	HEX											GRAMM			Rosetta
	run1	run2			run3							run2			run3
structure ID	1	**12**	**28**	37	3	18	22	32	42	44	46	2	3	5	2
Surface Connections	48	**148**	**134**	101	134	90	122	48	90	98	112	38	40	33	13
H bonds	2	**9**	**7**	5	7	4	8	3	4	3	3	3	3	2	4
Potential H bonds	6	**18**	**8**	9	8	10	10	5	10	8	10	2	2	1	2
salt bridges	0	**5**	**5**	4	5	3	2	0	2	2	0	0	0	0	2

**Table 7:** The distances (in Å) of Ca^2+^ ions after the minimization of the H1s1-H1s2-H2s2 trimer.

	Before minimization (Å)	After minimization (Å)	Suggested by Lodish (Å)
H1s1-H1s2	2.4	2.3	2.2
H1s1-H2s2	2.2	1.9	1.5
H1s2-H2s2	2.6	2.5	2.5

**Table 8:** Superficial attributes computed for the protei interfaces of the H1s1-H1s2-H2s2 trimer for each optimized trimer-ligand complex and agreement (R2) with experimental data.

Contributions	R^2^	Tri-antennary ligands				Bi-antennary ligands		
		compl1a	compl1	compl2	compl3	compl4	compl5	compl6
interface Acc. Surface area	0.62	4573.66	4236.97	3691.02	4102.78	3794.87	3851.59	3654.59
% interface Acc. Surface area	0.62	68.51	63.76	55.29	61.57	56.81	57.74	54.63
Planarity	0.46	9.79	9.37	8.12	8.9	8.44	8.15	8.61
Length/Breadth Ratio	0.09	3.54	3.46	3.38	3.98	3.43	3.69	3.61
Interface Residue Segments	0.63	27	28	25	27	26	26	24
% Polar Atoms in Interface	0.24	260.8	265.22	253.81	265.13	244.19	266.1	241.28
% Non-Polar Atoms in Interface	0.24	339	334.6	345.8	334.6	355.4	333.6	358.4
Hydrogen Bonds	0.20	28	22	20	30	20	20	20
Salt Bridges	0.23	2	2	0	4	0	0	0
Gap volume	0.55	19793.86	19735.54	20097.12	19910.5	19233.98	20622	20948.58
Gap volume index	0.73	16.68	18.78	21.84	18.04	20.92	21.92	24.64
